# Femtosecond-Laser Nanostructuring of Black Diamond Films under Different Gas Environments

**DOI:** 10.3390/ma13245761

**Published:** 2020-12-17

**Authors:** Marco Girolami, Alessandro Bellucci, Matteo Mastellone, Stefano Orlando, Valerio Serpente, Veronica Valentini, Riccardo Polini, Elisa Sani, Tilde De Caro, Daniele M. Trucchi

**Affiliations:** 1 DiaTHEMA Lab, Istituto di Struttura della Materia, Consiglio Nazionale delle Ricerche (ISM–CNR), Sede Secondaria di Montelibretti, Via Salaria km 29,300, Monterotondo Stazione, 00015 Roma, Italy; alessandro.bellucci@ism.cnr.it (A.B.); matteo.mastellone@ism.cnr.it (M.M.); valerio.serpente@ism.cnr.it (V.S.); veronica.valentini@ism.cnr.it (V.V.); daniele.trucchi@ism.cnr.it (D.M.T.); 2 Dipartimento di Scienze di Base ed Applicate per l’Ingegneria, Università di Roma “La Sapienza”, Piazzale Aldo Moro 5, 00185 Roma, Italy; 3 Istituto di Struttura della Materia, Consiglio Nazionale delle Ricerche (ISM–CNR), Sede Secondaria di Tito Scalo, Area Industriale–Contrada S. Loia, Tito Scalo, 85050 Potenza, Italy; stefano.orlando@ism.cnr.it; 4 Dipartimento di Scienze e Tecnologie Chimiche, Università di Roma “Tor Vergata”, Via della Ricerca Scientifica 1, 00133 Roma, Italy; polini@uniroma2.it; 5 Istituto Nazionale di Ottica, Consiglio Nazionale delle Ricerche (INO–CNR), Largo E. Fermi, 50125 Firenze, Italy; elisa.sani@ino.it; 6 Istituto per lo Studio dei Materiali Nanostrutturati, Consiglio Nazionale delle Ricerche (ISMN–CNR), Via Salaria km 29,300, Monterotondo Stazione, 00015 Roma, Italy; tilde.decaro@ismn.cnr.it

**Keywords:** femtosecond laser, diamond, black diamond, nanostructures, optical properties, Raman spectroscopy, solar absorptance

## Abstract

Irradiation of diamond with femtosecond (fs) laser pulses in ultra-high vacuum (UHV) conditions results in the formation of surface periodic nanostructures able to strongly interact with visible and infrared light. As a result, native transparent diamond turns into a completely different material, namely “black” diamond, with outstanding absorptance properties in the solar radiation wavelength range, which can be efficiently exploited in innovative solar energy converters. Of course, even if extremely effective, the use of UHV strongly complicates the fabrication process. In this work, in order to pave the way to an easier and more cost-effective manufacturing workflow of black diamond, we demonstrate that it is possible to ensure the same optical properties as those of UHV-fabricated films by performing an fs-laser nanostructuring at ambient conditions (i.e., room temperature and atmospheric pressure) under a constant He flow, as inferred from the combined use of scanning electron microscopy, Raman spectroscopy, and spectrophotometry analysis. Conversely, if the laser treatment is performed under a compressed air flow, or a N_2_ flow, the optical properties of black diamond films are not comparable to those of their UHV-fabricated counterparts.

## 1. Introduction

In the field of concentrated solar energy conversion, black diamond is gaining increasing attention, thanks to its outstanding solar absorptance values, even exceeding 90% [1,2,3], as well as for its suitability for operation at very high temperatures in thermionic emission-based devices [4,5,6]. “Black” diamond is obtained by a surface treatment of native semi-transparent diamond with linearly-polarized ultrafast pulsed laser beams, which induce the formation of nanometric periodic structures (LIPSS, Laser Induced Periodic Surface Structures [7]) able to strongly enhance the interaction between material and sunlight. Even if LIPSS can been successfully obtained with nanosecond (ns) [8] laser pulses, the use of ultrafast pulse duration regimes, such as picosecond (ps) or, even better, femtosecond (fs), allows for a more precise control of LIPSS geometry, because thermal effects are significantly reduced.

In recent years, lots of research activities have been reported on LIPSS, which represent a versatile and practical way to functionalize the surface of metals [9,10,11,12,13], dielectrics [14,15,16] and semiconductors [17,18,19]. Current applications of LIPSS span from the control of surface wetting to the tailoring of surface colonization by bacterial biofilms, from the improvement of the tribological performance of nanostructured metal surfaces to the efficient water-oil separation in marine oil spills [20]. LIPSS allows indeed for an accurate control of the physical and/or chemical properties of the laser-treated materials. In particular, in the case of wide-bandgap semiconductors (e.g., diamond [1,2,3,4,5,6] and SiC [21,22]), surface fs-laser treatments, leading to the formation of LIPSS, strongly influence the optical properties, increasing solar absorptance, in two different ways. First, LIPSS acts as a diffraction grating for the impinging photons, thus enhancing light trapping [23]; in this sense, it is crucial to ensure regular, well-defined structures uniformly distributed over the largest possible area of the treated material, aimed at minimizing the escape probability of coupled light. Moreover, by unavoidably creating defects, fs-laser treatments always introduce energy levels within the semiconductor bandgap, which are eventually responsible for photon absorption [24]. In a few words, in black diamond films for solar applications, LIPSS-induced light trapping increases the optical path length (i.e., the distance that an unabsorbed photon may travel within the material before escaping out), thus increasing the probability for solar photons to be absorbed by sub-bandgap defect-related energy levels introduced by the laser treatment.

The key control parameter for the fs-laser treatment of wide-bandgap semiconductors is the total accumulated laser fluence (*Φ_A_*, for a more detailed description see Section 2), which can be roughly defined as the “dose” of energy deposited into the material per surface unit. A low value of *Φ_A_* results in incomplete LIPSS formation, which reflects both into a poor light trapping and a too low density of defects to enhance sub-bandgap photon absorption. By increasing *Φ_A_*, the formation of better-defined LIPSS is observed, until an optimal *Φ_A_* is reached, corresponding to regular one-dimensional (1D) periodic structures with a high degree of structural integrity, that ensures excellent light trapping capabilities and solar absorptance values around 90% in the case of black diamond [2,6]. Higher values of *Φ_A_* result in a progressive degradation of the structural quality of the induced LIPSS; yet despite this, sub-bandgap photon absorption is further enhanced of a few percent [2,6] with respect to the case of optimal *Φ_A_*, because the poorer light trapping capability is over-compensated by a higher density of defect-related energy levels within the semiconductor bandgap. For the fabrication of a reliable device for concentrated solar energy conversion, however, an optimal *Φ_A_* should always be pursued for two reasons: (1) it ensures reproducible LIPSS with reproducible geometric features; (2) the slightly lower optical absorption capability with respect to highly defected LIPSS obtained at higher *Φ_A_* is compensated by better electronic properties in terms of transport of photogenerated charge carriers, which is particularly important for active devices requiring adequate quantum efficiency (i.e., the ability of converting absorbed photons into an exploitable current) [6,24].

Up to now, femtosecond-laser nanostructuring of black diamond films has always been performed in ultra-high vacuum (UHV) chambers, mainly because UHV conditions avoid the exposure of the surface under treatment to possible contaminants or reactive species. However, the use and maintenance of complex UHV systems conflict with the cost-effectiveness of black diamond technology, and do not represent the best option for a possible future large-scale production of devices for solar concentrating systems. For this purpose, aimed at an easier manufacturing workflow of black diamond, we performed for the first time fs-laser treatments on polycrystalline diamond samples at ambient conditions (i.e., room temperature and atmospheric pressure) under different gas flows: compressed air, N_2,_ and He. In a recent work [25] it was reported that well-defined LIPSS, uniformly distributed over a large area can be obtained on single-crystal diamond samples fs-laser treated in air. Conversely, in this work we demonstrate that treatments in air, as well as under a N_2_ flow, are not the optimal solution for polycrystalline diamond samples, whereas the use of a He flow ensures the fabrication of black diamond films with optical properties comparable to those obtainable with UHV treatments.

## 2. Materials and Methods

Three freestanding 10 × 10 × 0.3 mm^3^ polycrystalline CVD (Chemical Vapour Deposition) “thermal management (TM) grade” diamond samples, provided by Element Six Ltd. (Didcot, UK), were selected for the present work. For convenience, the three samples were labelled as TM1, TM2, and TM3.

Before starting the laser treatments, all the samples were subjected to a standard cleaning procedure, aimed at removing possible non-diamond contents, as well as residual contaminants derived from the polishing procedure. Diamond plates were dipped in a strongly oxidizing mixture (Carlo Erba Reagents S.r.l., Cornaredo, Milan, Italy), composed by HNO_3_ (purity 70%), H_2_SO_4_ (purity 96%), and HClO_4_ (purity 71%) in the 1:1:1 volume ratio, for 15 min at boiling point. Subsequently, the samples were subjected to ultrasound cleaning in acetone for 5 min, then in 2-propanol for a further 5 min. Samples were finally rinsed in deionized water.

**Figure 1:**
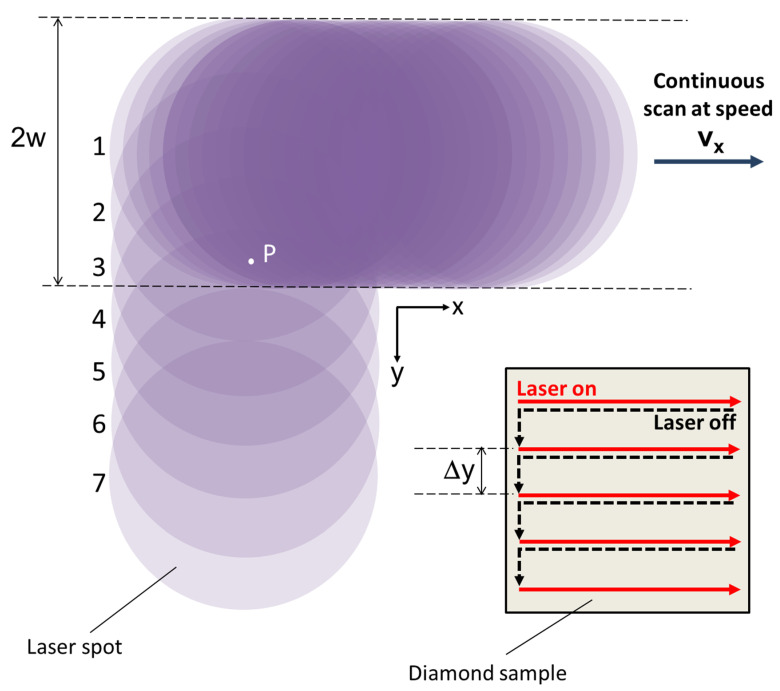
Laser scanning patterns used for the treatment of the diamond samples. The laser beam scans the sample (“laser on”) along *x*, than blanks (“laser off”), moves back to the starting point, shifts along *y* by a distance Δ*y*, switches on again, and starts a new scan along *x*. For the sake of clarity, only the first laser spot of horizontal scans #2 to #7 is shown. Note that the point P is subjected to five laser scans along *x* (rows 1 to 5), but it is not irradiated when the laser scans rows 6 and 7.

Laser processing sessions were performed at room temperature and atmospheric pressure with a linearly polarized fs-pulsed laser beam (wavelength *λ*_fs_ = 800 nm), generated by a mode-locked regeneratively amplified “chirped-pulse” Ti:sapphire laser system. Repetition rate, *f*, was set to 1 kHz. Pulse duration was set to ~100 fs. Diamond samples were placed on an *x*-*y* translational stage with micrometric resolution and automatic control capabilities (Laser μFAB microfabrication Workstation, from Newport, Irvine, CA, USA). For every diamond plate, the laser beam, with a spot-size of diameter (1/*e*^2^ width) equal to 2*w* = 150 µm, scanned a delimited surface area (namely, a 8 × 8 mm^2^ square) according to a raster pattern. Figure 1 reports a sketch of the scanning process; as can be visualized, the laser beam sweeps horizontally left-to-right at a constant speed, *v*_x_, then switches off and quickly moves back to the starting point; after that, it shifts vertically by a distance Δ*y*, switches on again, and starts a new scan along *x*-axis. The sequence is repeated until a 8 × 8 mm^2^ square is processed. For a single scan along the *x*-axis, the accumulated horizontal laser fluence, *Φ_x_*, is defined as *Φ_x_ = N_x_Φ_p_,* where *N_x_* is the average number of pulses irradiating the surface unit (exemplified by the point P in Figure 1), and *Φ_p_ = E_p_*/(*πw*^2^) is the single pulse laser fluence, with *E_p_* and *w* being the pulse energy and the radius of the circular laser spot on the focal plane, respectively. However, to obtain the total accumulated laser fluence delivered to the surface unit, *Φ_A_*, we also have to take into account the vertical overlap Δ*y* between two consecutive horizontal scans, i.e., *Φ_A_ =* (2*w/*Δ*y*) *Φ_x_*. In our case, we obtain *Φ_A_* = 7.5 *Φ_x_*, being Δ*y* = 20 μm and 2*w* = 150 μm.

The same value of total accumulated laser fluence was delivered to each of the treated samples. In particular, we chose *Φ_A_* = 5.0 kJ/cm^2^, corresponding to the best periodic 1D structures (in terms of structural integrity and geometric regularity) obtained in UHV-fabricated black diamond samples of the same crystalline grade, as previously reported [2]. In all the different laser treatments, the single pulse energy was kept constant. More precisely, it was set to *E_p_* = 785 μJ, which corresponds to a single pulse laser fluence *Φ_p_* = 4.44 J/cm^2^, which is slightly above the ablation threshold for polycrystalline CVD diamond, estimated to be about 3 J/cm^2^ with a single 100 fs pulse irradiation at 800 nm [26]. A constant value of single pulse laser fluence allowed us to adjust the accumulated horizontal laser fluence *Φ_x_* by simply varying the laser scanning speed *v*_x_, which, together with the repetition rate *f*, determines the average number of pulses *N_x_* irradiating the surface unit through the relationship *N_x_* = 2*wf/v*_x_. By choosing *v*_x_ = 1 mm/s, which represents a good trade-off between effectiveness and speed of the laser treatment, we obtained *N_x_* = 150 and *Φ_x_* = 0.67 kJ/cm^2^, resulting in the desired value of total accumulated laser fluence *Φ_A_* = 5.0 kJ/cm^2^.

Throughout the duration of the laser treatment, a constant gas flow was directed towards the sample surface. Aimed at ensuring the same exposure conditions on the whole surface, the gas flow direction formed an angle with the surface normal very close to 90°, so to avoid the formation of a boundary layer with a spatially dependent thickness. Therefore, the only experimental condition which was varied from one treatment session to another was the type of gas used: compressed air (standard ISO 8573-1:2010 [27], purity class 1) for TM1, N_2_ (purity > 99.998%) for TM2, and He (purity > 99.5%) for TM3. After the treatments, all the sample were subjected to the same cleaning procedure previously described (HNO_3_:H_2_SO_4_:HClO_4_ = 1:1:1, 15 min at boiling point), aimed at removing debris caused by the ablation process.

The morphology of the fabricated black diamond samples was evaluated with a “Leo-Supra35” field emission scanning electron microscope (FE-SEM) (Zeiss, Oberkochen, Germany). Prior to starting SEM characterization, an ultra-thin (~2 nm) Au coating was deposited on the surface of each sample by DC sputtering to avoid sample charging. After the end of SEM analysis, the Au coating was simply removed by dipping the samples in *aqua regia* (HNO_3_:HCl in the 1:3 volume ratio, 5 min at boiling point), then samples were rinsed in deionized water.

Aimed at investigating the laser-induced structural modifications, a comprehensive Raman characterization was successively performed on the black diamond samples. Raman spectra in the 1000–1800 cm^−1^ range were acquired at room temperature and in back-scattering geometry with an Ar^+^ laser (514.5 nm wavelength). A Dilor XY triple spectrometer (Dilor Instruments SA, Edison, NJ, USA) was used, equipped with an Olympus confocal microscope (Olympus Corporation, Tokyo, Japan) and a liquid nitrogen-cooled charge coupled device (CCD) detector. The laser spot-size was set to 2 µm. Data were then processed with Thermo-Grams Suite v9.2 software (Thermo Fisher Scientific Inc., Waltham, MA, USA), aimed at calculating widths and intensities of the Raman peaks. In particular, peak profiles were fitted by using Voigt functions.

The optical properties of nanotextured black diamond samples were finally investigated by spectrophotometric analysis, aimed at evaluating their absorptance in the solar wavelength range, whereas a detailed defect spectroscopy study is beyond the scope of this work. Room temperature spectrophotometry in the wavelength range 0.25–2.5 μm was performed by measuring optical transmittance (*τ*) and hemispherical reflectance (*ρ*) with a “Lambda900” double-beam spectrophotometer (PerkinElmer, Waltham, MA, USA) equipped with a Spectralon^®^-coated integrating sphere (150 mm diameter). Spectra were acquired at quasi-normal incidence conditions. Absorptance (α) was obtained from transmittance and reflectance values through the conservation of energy relationship [28]:

α + *τ* + *ρ* = 1 (1)

## 3. Results

In the following subsections, the results of the morphological, structural, and optical characterization of the three black diamond samples (TM1, TM2, TM3) fabricated under different gas environments (air, N_2_, He, respectively) will be introduced and compared to those previously reported on their UHV-fabricated counterparts [2,3].

### 3.1. SEM Characterization

**Figure 2:**
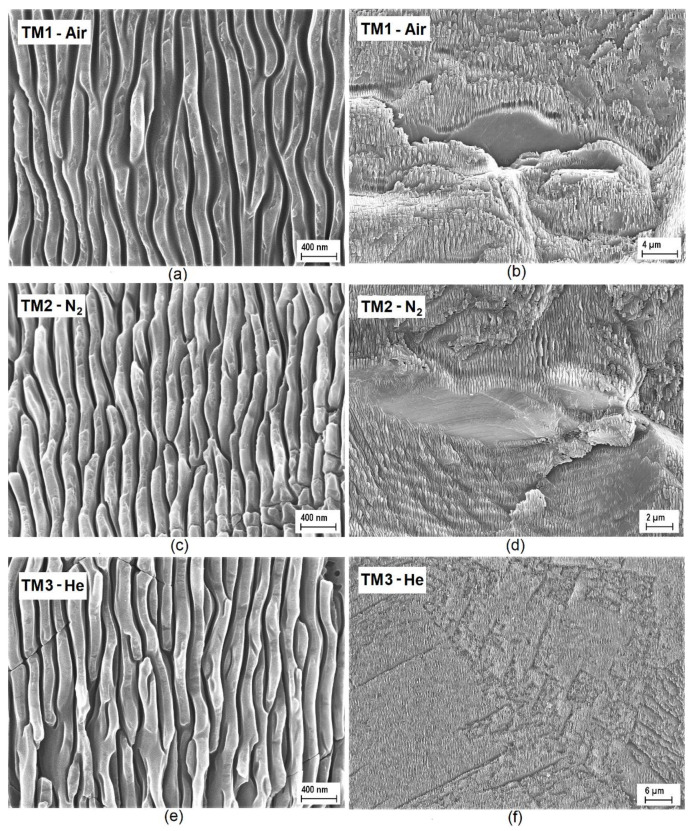
Higher magnification (left) and lower magnification (right) SEM images of the surface of samples TM1, TM2, and TM3, laser-treated under a compressed air (**a**,**b**), a N_2_ (**c**,**d**) and a He (**e**,**f**) flow, respectively.

The left side of Figure 2 shows three high-magnification SEM images taken from the surface of the three nanostructured black diamond films. As can be seen, no significant difference in LIPSS morphology can be highlighted among the samples treated under a compressed air (Figure 2a), a N_2_ (Figure 2c), or a He (Figure 2e) flow: in all cases, well-formed and rather regular LIPSS can be noticed, with a periodicity, Λ, of about 170 ± 10 nm, which perfectly matches the theoretical value of 166 nm given by the relationship Λ = *λ*_fs_/2*n*, where *n* = 2.41 is the refractive index of diamond at the laser wavelength *λ*_fs_ = 800 nm used for the treatments [29]. LIPSS depth, as inferred from SEM images recorded in tilted configuration, was found to be about 480 ± 20 nm. Periodic structures well resemble those obtained on UHV-fabricated black diamond films of the same crystalline quality, treated at the same laser wavelength, and with the same value of total accumulated laser fluence [2]. So we can conclude that the gas environment of the laser treatment has little influence on the morphological and geometrical features of LIPSS.

**Figure 3:**
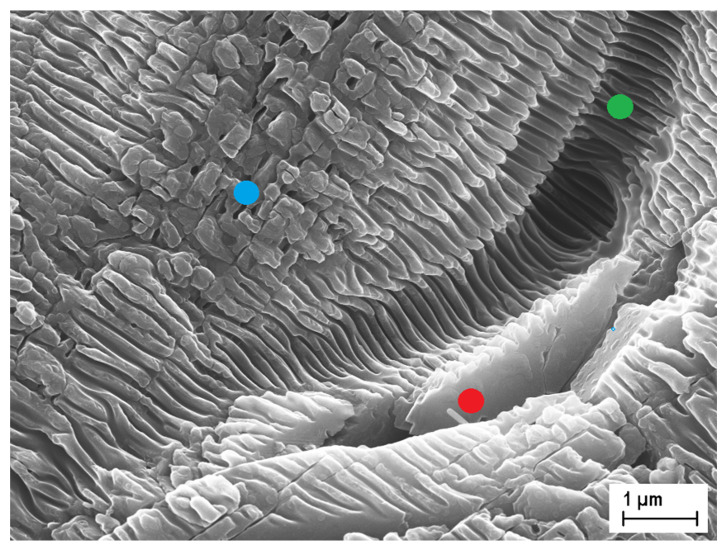
High-magnification detail of the step-terraced surface of sample TM1-air, laser-treated under a compressed air flow. LIPSS are present in most of the treads (green dot), but sometimes they are missing (red dot), or degenerate into irregular nano-squares (blue dot).

Conversely, looking at the right side of Figure 2, which shows representative lower magnification SEM images of larger areas of the laser-treated samples, it can immediately be seen that the surfaces of the samples treated under a compressed air (Figure 2b) or a N_2_ flow (Figure 2d) differ significantly from the surface of the sample treated under a He flow (Figure 2f). In the case of TM1-air and TM2-N_2_, surfaces have indeed a terraced morphology, with irregularly shaped treads and risers of different heights, whereas TM3-He has a substantially flat surface, with shallow scratches most probably caused by the mechanical polishing procedure performed on the untreated sample. Most significantly, while LIPSS are rather uniformly distributed throughout the whole surface of TM3-He, as in the case of a UHV-fabricated black diamond film, the same cannot be said for TM1-air and TM2-N_2_ samples. As can be more clearly seen from Figure 3, showing a detail of the surface of TM1-air (but the same considerations can be made for TM2-N_2_), most of the treads show regular LIPSS (green dot), but in some cases LIPSS degenerate into irregular square-like nanostructures (blue dot), or they do not even form at all (red dot).

A possible explanation for the irregular morphology of TM1-air and TM2-N_2_ surfaces, as well as for the poor uniformity of LIPSS distribution, can be given if we observe that deep cracks (see Figure 2b,d) and grooves (see Figure 3) do not follow the laser scanning direction, but rather randomly-oriented curved paths, which can extend for several tens of micrometers, i.e., the typical grain size of a CVD “thermal management grade” polycrystalline diamond sample with a thickness of a few hundreds of micrometers [30]. Therefore, such curved paths run most likely along the grain boundaries, which are the preferential incorporation sites of nitrogen [31]. Our hypothesis is that when the Gaussian laser beam, during the raster scan process, irradiates with its tails (i.e., at a fluence between the modification threshold and the ablation one), a grain boundary exposed to a compressed air or a N_2_ flow, incorporation of nitrogen is strongly enhanced by local heating. This leads to bond distortions [32], induces a significant mechanical stress in the surrounding lattice, and increases the number of vacancy defects [33]. As a consequence, the ablation threshold decreases in correspondence of a grain boundary, as well as in its neighborhood, so that the subsequent full-spot irradiation (i.e., at the peak fluence) results in a much higher ablation rate. A high fluence ablation regime [34] may indeed be triggered, with the formation of deep cracks and the ejection of differently sized shivers [35], which are then re-deposited as debris or carried away with the gas flow. Such re-deposited shivers, by partially masking the sample surface from laser irradiation, may be responsible for the presence of untreated areas in TM1-air and TM-N_2_ samples (see Figure 2b,d and Figure 3).

It is worth stressing here that femtosecond laser treatments performed in air on high-quality single crystal diamond plates result in regular LIPSS uniformly distributed over the whole sample surface, which substantially retains its original flatness without the formation of deep cracks and/or terraces [25]. This may support our hypothesis on the role that grain boundaries play in hampering a reproducible and reliable fabrication process of black diamond films in air or in a N_2_-rich environment. A He environment, conversely, ensures a uniform treatment over large areas even on polycrystalline diamond samples.

### 3.2. Raman Characterization

**Figure 4:**
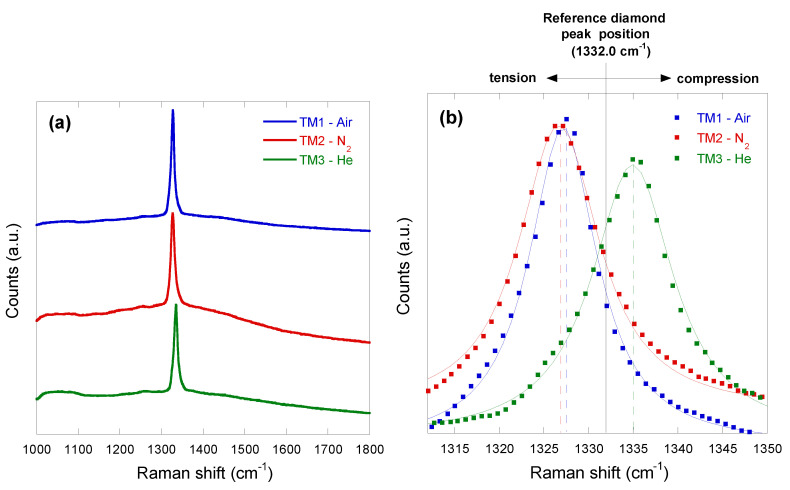
(**a**) Full Raman spectra of the three black diamond films fabricated under different gas environments. Spectra were vertically shifted for the sake of clarity; (**b**) Details of the three spectra shown in Figure 4a in the Raman shift range around the reference diamond peak position at 1332.0 cm^−1^; square dots represents raw data, solid lines indicate the Voigt functions used to fit the peak profiles, whereas dashed lines indicate the diamond peak position of the three different black diamond films.

Figure 4a shows the Raman spectra of the three fabricated black diamond films in the full analyzed range 1000–1800 cm^−1^. As can be seen, the only feature appearing in all the spectra is a sharp strong line centered at about 1332 cm^−1^, which is the well-known first-order peak related to crystalline diamond. As in the case of an untreated diamond sample, as well in those of all the black diamond films laser-treated in UHV at different values of total accumulated fluence, no other carbon-related feature (e.g., graphite, diamond-like carbon) is present. Linewidths (Full Width Half Maximum, FWHM) are all in the 7–10 cm^−1^ range regardless of the gas environment, thus partially overlapping the 4–8 cm^−1^ range measured on their UHV-fabricated counterparts [2]. In black diamond films, the large FWHM value of the 1332 cm^−1^ peak, always exceeding 4–5 cm^−1^ in the case of polycrystalline samples, is due to the laser-induced creation of intra-grain defects, which degrade the structural quality of crystalline grains.

Most interestingly, the gas environment of the laser treatment was found to strongly influence the Raman shift position of the diamond peak, which always differs from the reference value of 1332.0 cm^−1^. In particular, diamond peak positions were measured to be 1326.3 cm^−1^, 1326.9 cm^−1^, and 1335.1 cm^−1^ for TM1-air, TM2-N_2_, and TM3-He, respectively. This implies that samples treated under a compressed air flow or a N_2_ flow are subjected to approximately the same amount of tensile stress, whereas the sample treated under a He flow is subjected to a compressive stress. It is worth mentioning here that an upshifted diamond Raman peak, denoting a compressive stress as in the case of TM3-He, has been observed in all the UHV-fabricated black diamonds, showing the best LIPSS in terms of geometrical regularity and structural integrity [2,3,6,24]. Conversely, a downshifted diamond Raman peak, denoting a tensile stress as in the case of TM1-air and TM2-N_2_, has only been observed in “thermal management grade” black diamond plates UHV-treated at high values of total accumulated laser fluence (>10 kJ/cm^2^), which resulted in a very damaged and irregular surface [2,24].

### 3.3. Spectrophotometric Analysis

**Figure 5:**
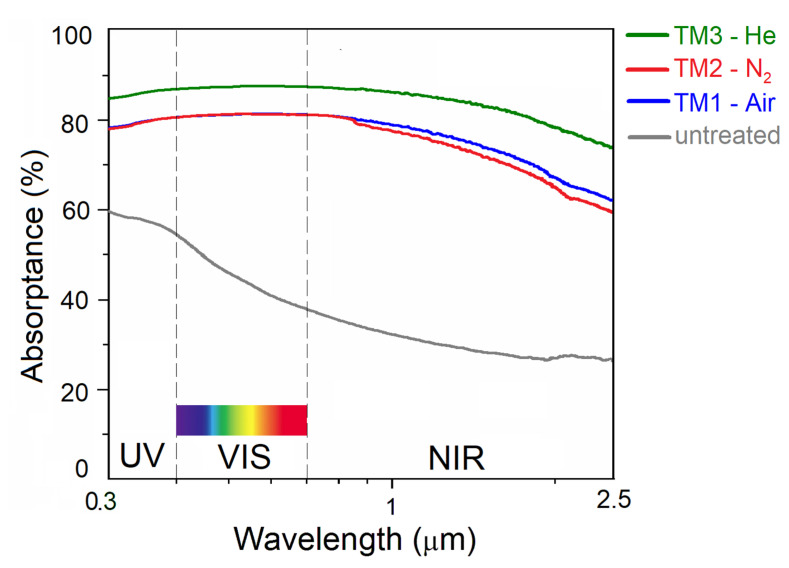
Absorptance spectra of the three black diamond films laser-treated under different gas environments, obtained by spectrophotometric analysis according to Equation (1). For comparison, the absorptance spectrum of an untreated sample (gray curve) with the same crystalline quality is shown.

Figure 5 shows the absorptance spectra of the three fabricated black diamond samples, as well as of a reference untreated sample of the same crystalline quality. Noticeable is the increase of absorptance in the whole wavelength range for all the treated samples with respect to the reference diamond, thus validating the effectiveness of all the fabrication processes at room temperature and atmospheric pressure under different gas environments. As expected from the SEM and Raman characterizations, showing very similar results for TM1-air and TM2-N_2_, absorptance curves measured for the samples treated under a compressed air or a N_2_ flow are almost superimposable, with slightly higher values for TM1-air in the near-infrared (NIR) region.

Most significantly, TM3-He, laser-treated under a He flow, shows absorptance values around 85% in the ultraviolet (UV) range, between 85 and 90% in the visible (VIS) range, and between 80% and 90% in the NIR range: this curve retraces almost perfectly the absorptance spectrum measured for a polycrystalline black diamond sample of the same crystalline quality UHV-treated at the same *Φ_A_* value (5.0 kJ/cm^2^) [2,3]. The solar absorptance, α_S_, of the different black diamond films can be obtained from the following equation:

(2) $$\alpha_{S}=\frac{\int_{\lambda \;=\;200\;nm}^{\lambda \;=\;2.0\;\mu m}\alpha \left(\lambda \right)W\left(\lambda \right)d\lambda }{\int_{\lambda \;=\;200\;nm}^{\lambda \;=\;2.0\;\mu m}\;W\left(\lambda \right)d\lambda }$$

where *α*(*λ*) is the absorptance as a function of the exciting wavelength *λ*, and *W*(*λ*) is the global-tilt (GT) 1.5 air mass (AM) solar irradiance [36,37]. Calculated α_S_ values, according to Equation (2), are 79.1%, 78.4%, and 86.2% for TM1-air, TM2-N_2_ and TM3-He, respectively. In particular, the solar absorptance value obtained for TM3-He (86.2%) is very close to the one (88.4%) measured on its UHV-treated counterpart [2], therefore confirming that the use of a He flow at ambient conditions is a valid alternative to a UHV chamber for the fabrication of polycrystalline black diamond films with enhanced optical absorption properties.

## 4. Discussion

As mentioned in the introductory section, absorptance enhancement in black diamond samples is both due to a more effective light trapping and the introduction of defect-related energy levels within the bandgap, which promote sub-bandgap photon absorption. The geometrical quality and structural integrity of the laser-induced LIPSS has of course a strong influence on light trapping, because a well-defined diffraction grating, uniformly distributed over the sample surface, ensures an efficient coupling of impinging photons. Conversely, as it is easy to deduce, more defected LIPSS reflect into a larger density of defect-related sub-bandgap energy levels.

Now, by observing the higher magnification SEM images of the LIPSS fabricated under the three different gas environments (Figure 2a,c,e), no substantial morphological differences can be noticed; therefore we can deduce that microscopically (i.e., within a LIPSS-containing domain), both the light trapping capability and the distribution of defect-related energy levels within the bandgap are approximately the same. What really marks the difference between the three cases can be easily inferred from the lower magnification SEM images, showing strongly irregular surfaces (alternating LIPSS-containing domains, flat zones, and deep grooves) for TM1-air (Figure 2b) and TM2-N_2_ (Figure 2d), and a uniform regular distribution of LIPSS for TM3-He (Figure 2f). As a result, an effective trapping of photons impinging all over the surface (as in the case of a UHV-treated sample) is only ensured for laser treatments performed under a He flow; this may be the reason of the increased absorptance of TM3-He (Figure 5) with respect to TM1-air or TM2-N_2_. The lack of significant discontinuities in the LIPSS spatial distribution may also explain why a compressive stress, typical of polycrystalline black diamond films UHV-treated at optimal *Φ_A_* values, is only present in TM3-He (Figure 2b), whereas TM1-air and TM2-N_2_ show a tensile stress typical of strongly damaged surfaces. Of course, the extended defects (e.g., deep grooves) of TM1-air and TM2-N_2_ may surely imply local enhancements of photon absorption by introducing a high density of sub-bandgap energy levels, but apparently this cannot compensate for the lack of a widely distributed uniform optical grating (as in the TM3-He case) able to increase light trapping efficiency.

In conclusion, while the total accumulated laser fluence is the key parameter to obtain LIPSS with high geometrical quality and structural integrity, the gas environment the laser treatment is performed within turns out to be crucial for the homogeneous distribution of LIPSS on the surface of the resulting black diamond film. From this point of view, laser treatments under a He flow are very effective, and are demonstrated to be the starting point for a cost-effective and reproducible fabrication of polycrystalline black diamond films with superior properties in terms of optical absorption. Directions for future research are based on a further refinement of the optimal value of the total accumulated laser fluence used for the treatments, aimed at a simultaneous enhancement of absorptance and quantum efficiency of the treated material. Moreover, the possibility of exploiting a He flow for implementing more complex nanostructures (e.g., two-dimensional LIPSS with enhanced light-trapping capabilities [38]) which have already been demonstrated with UHV treatments, will be explored.
